# Sphingosylphosphorylcholine (SPC), a Causative Factor of SPC-Induced Vascular Smooth Muscle Cells Contraction, Is Taken Up via Endocytosis

**DOI:** 10.3390/cells12020265

**Published:** 2023-01-09

**Authors:** Natsuko Tsurudome, Yuji Minami, Katsuko Kajiya

**Affiliations:** 1 The United Graduate School of Agricultural Sciences, Kagoshima University, 1-21-24 Korimoto, Kagoshima 890-0065, Japan; 2 Department of Food Science & Biotechnology, Faculty of Agriculture, Kagoshima University, 1-21-24 Korimoto, Kagoshima 890-0065, Japan

**Keywords:** endocytosis, exocytosis, fisetin, microdomains, sphingosylphosphorylcholine, vascular smooth muscle contraction

## Abstract

The reaction field of abnormal vascular contraction induced by sphingosylphosphorylcholine (SPC) and the action point of SPC around the plasma membranes remain unknown. However, we found in a previous study that fisetin prevents SPC-induced vascular smooth muscle cells contraction, while the mechanism remains unknown. Therefore, in this study, we aimed to address the action point of SPC around the plasma membranes and the involvement of fisetin. We focused on microdomains and evaluated their markers flotillin-1 and caveolin-1 and the localization of SPC to investigate their action point. The results showed that microdomains of vascular smooth muscle cells were not involved in SPC-induced contraction. However, we found that after SPC had been affected on the plasma membrane, cells took up SPC via endocytosis. Moreover, SPC remained in the cells and did not undergo transcytosis, and SPC-induced contracting cells produced exosomes. These phenomena were similar to those observed in fisetin-treated cells. Thus, we speculated that, although not involved in the reaction field of SPC-induced contractions, the microdomain induced the endocytosis of SPCs, and fisetin prevented the contractions by directly targeting vascular smooth muscle cells. Notably, this preventive mechanism involves the cellular uptake of SPC via endocytosis.

## 1. Introduction

Sphingosylphosphorylcholine (SPC) is a causative factor of abnormal vascular contraction, which can occur in blood vessels throughout the body. In particular, contractions in the brain or heart cause a variety of lethal diseases, such as cerebral infarction, angina pectoris, and myocardial infarction [1,2]. The vasculature is composed of intima, media, and adventitia, and smooth muscle cells composed of media are responsible for the contraction and relaxation of blood vessels. Notably, contractions can have two types of mechanisms: Ca^2+^-dependent normal contraction and Ca^2+^-independent abnormal contraction [3]. In a normal contraction, the cytosolic Ca^2+^ concentration in human coronary artery smooth muscle cells (HCASMCs) governs the activation of myosin light-chain (MLC) kinase. Additionally, contraction and relaxation are repeated by the reversible phosphorylation and dephosphorylation of MLC [4]. In contrast, activated Rho-kinase inactivates MLC phosphatase, inhibiting MLC dephosphorylation in SPC-induced contraction. This inhibits the relaxation of MLC [5], causing various diseases in the blood vessels. However, the mechanism underlying SPC-induced contraction remains unelucidated, making the development of therapeutic and preventive measures difficult. We have attempted to elucidate the mechanism of SPC-induced contraction. The phosphorylation of myosin phosphatase target subunit 1 (MYPT1) and MLC is also observed in Ca^2+^-dependent normal contractions. Therefore, it is difficult to distinguish between normal contraction and abnormal contraction. SPC is a lipid by-product of plasma membrane abnormal metabolism resulting from sphingomyelin (SM) [6] and a causative factor of abnormal contractions [7,8]. It stimulates HCASMCs and consequently activates Fyn tyrosine kinase and Rho-kinase in cells [9]. Previous studies have implicated the involvement of plasma membrane microdomains in SPC-induced contractions [10,11], as these contractions are induced based on the cholesterol content of vascular strips. Microdomains are highly functionalized local sites on the plasma membrane within the lipid bilayer, which are rich in cholesterol, sphingolipids, and functional proteins that comprise lipid rafts and caveolae [12]. Furthermore, they regulate cell proliferation and calcium signaling in HCASMCs [13]. However, their precise role and the metabolic pathway of SPC in SPC-induced contractions of HCASMCs are unclear. The cells stimulated with SPC do not relax again, leading to cell death. As previous reports suggesting the involvement of SPC receptors have been retracted, their identification has been extremely challenging. Therefore, determining the subcellular localization of SPC can help decipher the mechanism underlying SPC-induced contraction. 

We previously found that fisetin, a flavonoid found in several fruits and vegetables, prevents SPC-induced contractions of HCASMCs [14]. It affects HCASMCs directly by permeating vascular endothelial cells. However, the molecular mechanism underlying the preventive effects of fisetin in HCASMCs also remains to be elucidated.

Here, we explored the behavior of SPCs in abnormally contracting cells and the involvement of fisetin in preventive effects. Particularly, we investigated whether fisetin directly binds to SPC and how it functionally acts on HCASMCs. We also studied the intracellular uptake of SPCs produced upon plasma membrane metabolism and verified the previously implied role of microdomains in SPC-induced contractions.

## 2. Materials and Methods

### 2.1. Cell Culture

Normal HCASMCs (Kurabo, Osaka, Japan) recovered from a male were cultured in HuMedia-SG2 growth medium (Kurabo, Osaka, Japan) in a humidified atmosphere at 37 °C containing 5% CO_2_ (PHC, Tokyo, Japan) until 80–90% confluency. They were co-cultured with 1 μM of fisetin (Figure S1; Tokyo Kasei Kogyo, Tokyo, Japan) based on the methods of our previous study [14] after 24 h of cell seeding to determine the preventive effect of fisetin. Upon reaching confluency, the cells were cultured overnight in the basic medium HuMedia-SB2 (Kurabo, Osaka, Japan) and stimulated with 30 μM of SPC (Sigma Aldrich, St. Louis, MO, USA) for 10 min. SPC induces abnormal contraction of more than 30 μM [8,9,10,11,15,16,17]. Samples that exhibited the preventive effect of fisetin were stimulated with SPC in the presence of fisetin. Furthermore, we verified whether fisetin inhibited SPC directly by premixing fisetin and SPC and by stimulating the non-fisetin-treated cells with the premixed mixture. The microdomain was removed by treatment with methyl-β-cyclodextrin (MβCD; FUJIFILM Wako, Osaka, Japan) dissolved in HuMedia-SB2 and depleting cholesterol. SPC-induced contraction was evaluated according to previously reported methods [14,15,16]. Briefly, images of the cells were taken using an inverted microscope (CKX53, Olympus, Tokyo, Japan) for fluorescence imaging before and after the addition of SPC, and the cell surface area of the image was calculated by measuring the amount of Fluo3-AM using ImageJ ver. 1.52 software (NIH, Bethesda, MD, USA).

### 2.2. Live-Cell Imaging

We observed the morphology of SPC-induced contracting cells using the inverted microscope CKX53. Live-cell imaging was performed by first mounting HCASMCs onto glass coverslips, and staining the nuclei with DAPI (Sigma Aldrich, St. Louis, MO, USA) for >30 min after sample preparation, as mentioned in Section 2.1. Thereafter, we stained the plasma membranes with PlasMem Bright Red (Dojindo, Kumamoto, Japan) for 5 min or endosomes with 4 μM of FM4-64 (Sigma Aldrich, St. Louis, MO, USA), which can stain in live cells. The cells were then washed with HEPES buffer, and SPC-induced contraction was induced by 30 μM of nitrobenzoxadiazole (NBD)-SPC for 10 min; that is, SPC with guaranteed fluorescence, with NBD bound to the C-6 position of SPC (Cayman, Ann Arbor, MI, USA). Since SPC is a non-fluorescent compound, we evaluated its intracellular behavior using NBD-SPC, which is commonly used to study the metabolism and transport of sphingolipids [17]. Cell staining and cross-section images of cells were obtained using an all-in-one fluorescence microscope (BZ-X810, KEYENCE, Osaka, Japan) equipped with an optical sectioning module (BZ-H4XF, KEYENCE, Osaka, Japan). 

### 2.3. Flow Cytometry

Initially, we subjected the samples to the steps mentioned in Section 2.1. The cells were scraped, washed twice with 1% bovine serum albumin diluted in phosphate-buffered saline (PBS), and fixed in 1% paraformaldehyde in PBS. These cells were treated with NBD-SPC for 10 min at 37 °C and measured using BD FACSCalibur (BD Biosciences, Franklin Lakes, NJ, USA) or Attune NxT (Thermo Fisher Scientific, Waltham, MA, USA). Our gating strategy was to identify HCASMCs based on their size and granularity via forward scatter (FSC) and side scatter (SSC). Moreover, FSC/SSC measurement involved counting the number of contracted (Ec) and uncontracted (Euc) cells among the live-cell population. Contracted cell rates were calculated using the following formula:

Contracted cell rate (%) = Ec/(Ec+Euc) × 100

### 2.4. Surface Plasmon Resonance (SPR) Analysis

If the ligand and the analyte show 1:1 binding, the sensorgram can vary greatly with parameters such as pH, salt, and temperature of the solution. To avoid this in our study, we immobilized SPC on the surface of the Sensor Chip CM5 (GE Healthcare, Chicago, IL, USA) using a standard amine-coupling kit (Cytiva, Chicago, IL, USA). Fisetin (100 μM) was dissolved in dimethyl sulfoxide (Sigma Aldrich, St. Louis, MO, USA). As a positive control, RNA aptamer (GGGGAAAGCCUACCGUUAUUGGAGUAAAAACCCC) [18] (100 μM) dissolved in HBS (Cytiva, Chicago, IL, USA) was added over the chip surface as a ligand at a 10 μL/min flow rate at 25 °C, as described previously [19,20], to ensure that the reaction was performed under optimal conditions. All analyses were performed using Biacore T200 (Cytiva, Chicago, IL, USA), and the resultant data were analyzed using the Biacore T200 Evaluation Software 1.0 (Cytiva, Chicago, IL, USA) [19,20]. In SPR analysis, the sensorgram consists of an association and dissociation phase (Figure S2). The overall shape of the curve was determined from the kinetic analysis based on the analyte concentration and association and dissociation rate constants.

### 2.5. Western Blotting

The samples were lysed and fractionated into microdomains and other plasma membranes using the ULTRARIPA kit (BioDynamics, Tokyo, Japan), which can efficiently and quickly extract microdomains. These fractions were separated by 15% SDS-PAGE, eluted with 4× sample buffer (400 mM Tris pH 6.8, 8% SDS, 40% glycerol, 24% 2-mercaptoethanol, and 0.1% bromophenol blue), and transferred onto an Immun-Blot^®^ PVDF Membrane (Bio-Rad, Hercules, CA, USA). The membranes were blocked with 5% skim milk in Tris-buffered saline-Tween 20 for 60 min at 15–25 °C. Subsequently, they were probed with anti-caveolin-1 (1:1000, 610057, BD Biosciences, Franklin Lakes, NJ, USA), anti-flotillin-1 (1:1000, sc-133153, Santa Cruz Biotechnology, Santa Cruz, CA, USA), and anti-β-actin (1:5000, 5125S, Cell Signaling Technology, Danvers, MA, USA) antibodies. Thereafter, the membrane was incubated with anti-mouse Ig (1:5000, 554002, BD Biosciences, Franklin Lakes, NJ, USA), anti-mouse IgG (1:5000, sc-525408, Santa Cruz Biotechnology, Santa Cruz, CA, USA), and anti-rabbit IgG (1:5000, 7074S, Cell Signaling Technology, Danvers, MA, USA). The consequent protein bands were detected using the Clarity Western ECL Substrate and visualized on ChemiDoc XRS+ (Bio-Rad, Hercules, CA, USA).

### 2.6. Exosome Isolation and Detection

Exosomes were isolated from cell culture supernatant. The cells were cultured until 80–90% confluency was achieved, following which the medium was replaced with HuMedia-SB2 24 h before collecting the exosomes. The supernatant was centrifuged at 10,000× *g* for 30 min at 4 °C to remove the cells. Thereafter, it was ultracentrifuged at 213,863× *g* for 26 min at 4 °C in the Optima-TL and TLS-55 swinging-bucket rotor (Beckman Coulter, Brea, CA, USA) [21,22]. This was repeated until all the cell culture supernatants were eliminated. The pellets were washed with HEPES buffer in the same way. Exosome-containing pellets were stained with fluorescein isothiocyanate-labeled CD9 (312103, BioLegend, San Diego, CA, USA) and allophycocyanin-labeled CD63 (353007, BioLegend, San Diego, CA, USA), and were analyzed using BD FACSCalibur. Eventually, CD9 and CD63 exosomes were identified based on their size and granularity (FSC/SSC).

### 2.7. Statistical Analysis

Statistical analyses were performed using Microsoft Office Excel 2019. All experiments were performed independently at least thrice. Groups were compared using the Tukey–Kramer test for multiple comparisons with an α level of 0.05. Data are presented as mean ± standard deviation. 

## 3. Results

### 3.1. Fisetin Prevents SPC-Induced Contraction by Directly Acting on HCASMCs

**Figure 1:**
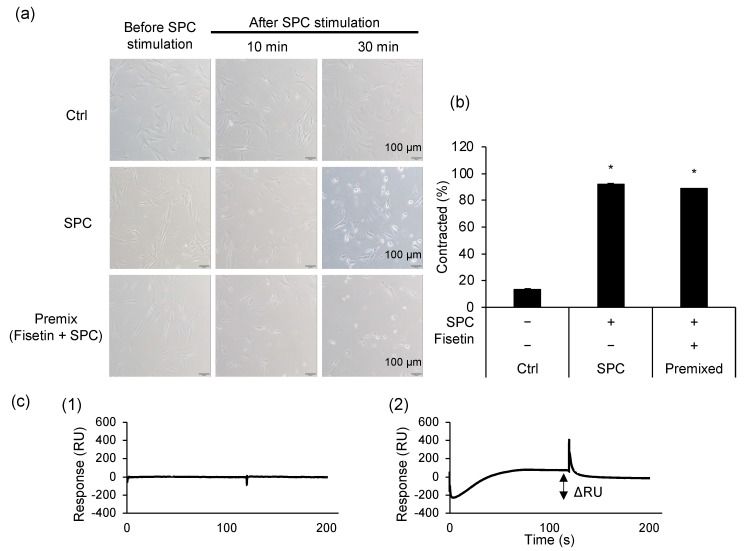
Fisetin does not interact directly with sphingosylphosphorylcholine (SPC), the causative factor of SPC-induced abnormal contraction. (**a**) Human coronary artery smooth muscle cells before and after SPC stimulation. Top row: control cells (Ctrl); middle row: SPC-stimulated cells; bottom row: SPC and fisetin premix-stimulated cells. The left column depicts cells before SPC stimulation, whereas the middle and right columns depict cells 10 or 30 min after SPC stimulation, respectively. (**b**) Contracted cell rates of Ctrl, SPC-, and premix-stimulated cells. Data are represented as mean ± standard deviation, * *p* < 0.05 vs. Ctrl. (**c**) Interaction between SPC and fisetin (1) or RNA aptamer (2). The solid line in the sensorgram indicates that fisetin does not interact with SPC, whereas the dashed line represents the binding curve of the RNA aptamer.

Fisetin prevents SPC-induced contractions by two possible mechanisms: it either directly binds to SPC, thereby obstructing SPC from acting on HCASMCs, or it directly acts on HCASMCs, altering their membrane structure or intracellular environment. Thus, we observed the morphology of HCASMCs and assessed the contracting cell rate. Additionally, molecular-to-molecular interactions were examined using SPR analysis to reveal whether fisetin directly binds to SPC. Although fisetin-treated HCASMCs prevent SPC-induced contraction (Figure S3 [14]), non-fisetin-treated cells stimulated with the premixed SPC and fisetin contracted identically to cells stimulated with SPC alone (Figure 1a). Remarkably, the calculated contracting cell rates supported these results (Figure 1b). In SPR analysis, the molecule-to-molecule interaction can be determined physicochemically from the association and dissociation curves, and the binding response shown in ΔRU depends on the strength of those interactions (Figure S2). The intermolecular interactions between SPC and fisetin also revealed that fisetin did not bind directly to SPC (ΔRU = 0), while the RNA aptamer did, the positive control bound to SPC (ΔRU = 351.8, Figure 1c). This was also in line with the quantification of fisetin in cells or the supernatant of fisetin-treated cells, in which almost all fisetin was in the cells (Figure S4).

### 3.2. Microdomains Are Not Essential for the SPC-Induced Contractions of HCASMCs

**Figure 2:**
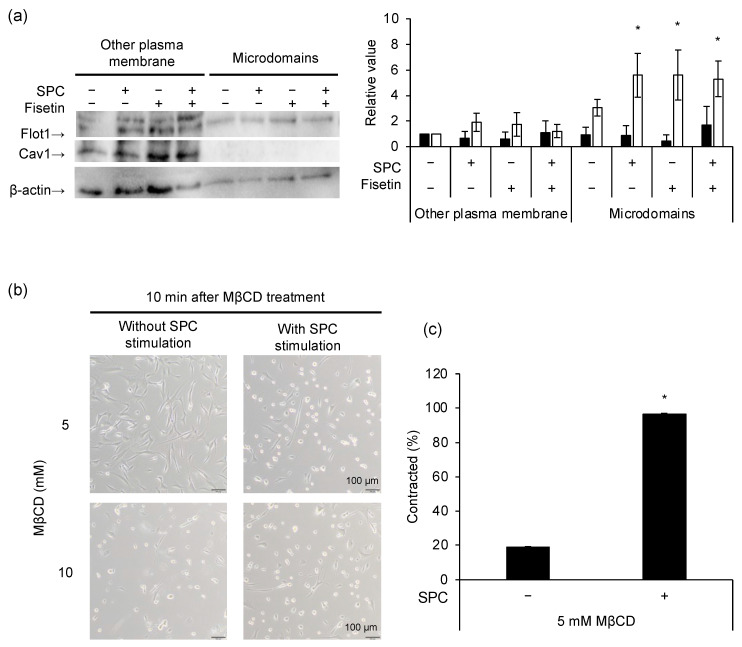
Analysis of sphingosylphosphorylcholine (SPC) and microdomain involvement in SPC-induced contractions of human coronary artery smooth muscle cells (HCASMCs). (**a**) Expression levels of the microdomain markers flotillin-1 (Flot1) and caveolin-1 (Cav1). Black and white indicate Cav1 and Flot1, respectively. (**b**) HCASMCs treated with 5 mM (top row) or 10 mM (bottom row) of methyl-β-cyclodextrin (MβCD). The left and right columns depict unstimulated and SPC-stimulated cells, respectively. (**c**) Contracted cell rates with microdomains disrupted by 5 mM of MβCD. * *p* < 0.05 vs. without SPC stimulation and fisetin treatment or only SPC stimulation. Data are represented as mean ± standard deviation.

We evaluated the involvement of microdomains in SPC-induced contraction by fractionating cells into microdomains and other plasma membranes. Additionally, we analyzed the expression levels of microdomain marker proteins, flotillin-1 (Flot1) and caveolin-1 (Cav1), via Western blotting. Consequently, we observed that Flot1 was expressed in the microdomain fraction, whilst both Cav1 and Flot1 were expressed in the other plasma membrane fraction (Figure 2a). Flot1 expression was upregulated upon SPC stimulation compared with that without SPC stimulation in the microdomains. However, fisetin-treated cells expressed more Flot1 than those without fisetin, even when not stimulated by SPC. This observation remained unchanged upon SPC stimulation. Thus, we evaluated the involvement of microdomains in SPC-induced contractions by disrupting the microdomains using MβCD, which removes cholesterol from the plasma membrane. The concentration and treatment time were assessed to verify the effect of the MβCD treatment. While HCASMCs were undamaged at 5 mM of MβCD without SPC stimulation, they contracted or peeled off for a short duration at 10 mM of MβCD despite the absence of SPC stimulation (Figure 2b, lower left). Therefore, we treated the cells with 5 mM of MβCD for 10 min and examined microdomain involvement by stimulating these cells with SPC. Consequently, SPC-induced contractions also occurred in MβCD-treated cells with microdomains removed (Figure 2b, upper right). Notably, the contracting cell rate was approximately 19.0% in unstimulated control cells and 96.7% in SPC-stimulated cells (Figure 2c).

### 3.3. SPCs Are Incorporated into Abnormally Contracting HCASMCs, Unrelated to Fisetin Treatment

**Figure 3:**
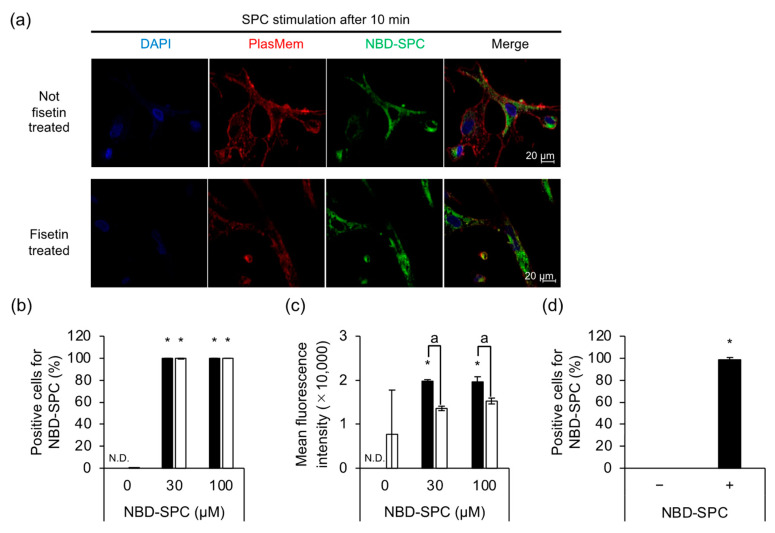
Localization of sphingosylphosphorylcholine (SPC) in human coronary artery smooth muscle cells (HCASMCs). (**a**) Representative image of NBD-SPC (green) localization in abnormally contracting HCASMCs. DAPI (blue), nuclear stain; PlasMem Bright Red (Red), plasma membrane stain. The top and bottom rows depict untreated control (Ctrl) cells and fisetin-treated cells. (**b**) Percentage of positive cells for NBD-SPC. Black and white indicate the number of abnormally contracting and non-contracting cells. (**c**) Mean fluorescence intensity of NBD-SPC. (**d**) Flow cytometric analysis of NBD-SPC-positive cells treated with 5 mM of MβCD. * *p* < 0.05 vs. 0 μM NBD-SPC-treated cells and a: *p* < 0.05 vs. abnormally contracting cells. N.D. = not detected. Data are represented as mean ± standard deviation.

We investigated the action point of SPC by observing their localization using live-cell imaging. NBD-SPC and SPC are interchangeable since NBD-SPC induces abnormal contraction. The results showed that NBD-SPCs were taken up into the cells regardless of fisetin treatment (Figure 3a). Our flow cytometry analysis also revealed that almost all HCASMCs were positive for NBD-SPC (Figure 3b). However, the mean fluorescence intensities differed between the abnormally contracting and non-contracting cells (Figure 3c). The percentage of SPC-induced contracting cells was 92.1%, whereas the remaining 7.9% contained non-contracting cells. Interestingly, MβCD-treated cells caused SPC-induced contraction in response to NBD-SPC as normal cells, regardless of the presence of microdomains (Figure 3d). This is supported by the results shown in Figure 2c.

### 3.4. Cellular Uptake of SPCs via Endocytosis

**Figure 4:**
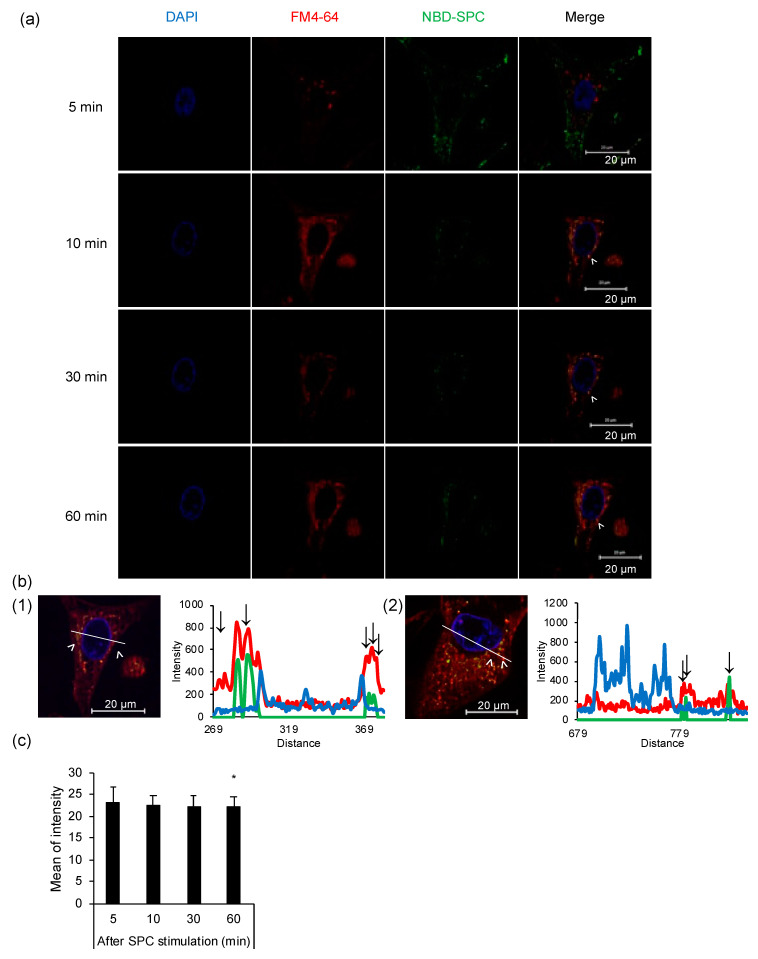
Cellular uptake of sphingosylphosphorylcholine (SPC). (**a**) Cellular uptake of nitrobenzoxadiazole (NBD)-SPC via endocytosis. Representative images indicate the localization of endosomes (red) and NBD-SPC (green) in the nucleus (blue). Top to bottom row: cells visualized 5, 10, 30, and 60 min after SPC stimulation. (**b**) The histogram shows the fluorescence profile for the enlarged image, illustrating the fluorescence intensity in each channel for endosomes (red line), NBD-SPC (green line), and the nucleus (blue line) against the distance along the line indicated on the micrograph. Arrows indicate co-localization of NBD-SPC and endosomes. SPC-stimulated human coronary artery smooth muscle cells (1) without fisetin and (2) with fisetin treatment. (**c**) Mean fluorescence intensity of cellular uptake of NBD-SPC after 5, 10, 30, and 60 min of SPC stimulation. Data are represented as mean ± standard deviation. * *p* < 0.05 vs. 5 min after SPC stimulation.

The localization of endosomes and SPCs was examined by live-cell imaging to confirm whether endocytosis is involved in SPC uptake into the cell. Five minutes after SPC stimulation, NBD-SPCs were still localized around the plasma membrane and not co-localized with endosomes (red and green did not overlap, Figure 4a). However, from 10 min onwards, NBD-SPCs co-localized with endosomes near the nucleus and could be observed continuously even after 60 min (red and green overlap). We assessed the intensities of each staining reagent in the cellular cross-sections and found that the NBD-SPC and FM4-64 peaks coincided, verifying the co-localization of NBD-SPC with endosomes. This phenomenon was also confirmed in fisetin-treated cells (Figure 4b). Subsequently, we evaluated whether SPC leaked out of the cells by assessing NBD-SPC intensity, and the intensity was unaltered for 30 min after SPC stimulation (Figure 4c and Figure S5). Therefore, we confirmed that SPC did not leak out of HCASMCs.

### 3.5. Exocytosis Is Caused by SPC-Induced Contractions but Is Unrelated to the Preventive Mechanism of Fisetin

**Figure 5:**
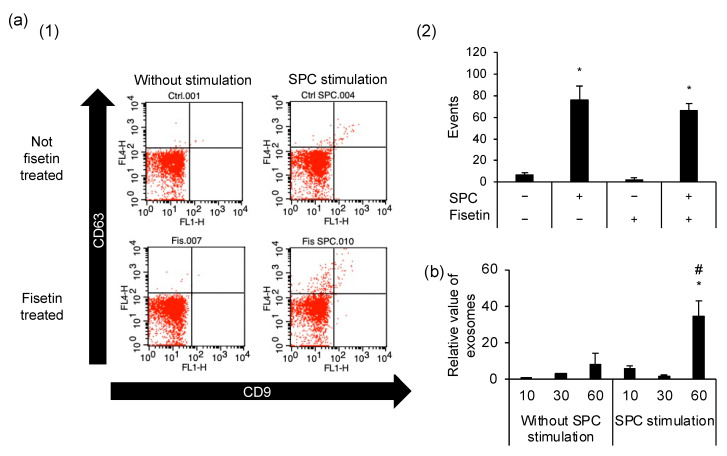
The number of exosomes in human coronary artery smooth muscle cells (HCASMCs). (**a**) (1) Flow cytometric dot plots representing CD9- and CD63-positive populations. (2) Number of exosomes generated from control (Ctrl) cells and fisetin-treated cells. (**b**) Number of exosomes in HCASMCs relative to unstimulated Ctrl cells. * *p* < 0.05 vs. Ctrl without stimulation. # *p* < 0.05 vs. without sphingosylphosphorylcholine (SPC) stimulation at the same time. Data are expressed as mean ± standard deviation.

We evaluated the effect of the SPC-induced contractions on exocytosis by analyzing the number of exosomes using flow cytometry. HCASMCs produced exosomes regardless of fisetin treatment and SPC stimulation; however, SPC-stimulated HCASMCs had a higher number of exosomes than unstimulated HCASMCs (Figure 5a). Furthermore, we discovered that the number of exosomes produced after SPC stimulation remained unaltered until 30 min, and it was higher in SPC-stimulated cells than in unstimulated cells after 60 min (Figure 5b).

## 4. Discussion

SPC is a lipid mediator generated from SM, which is a component of many cell types. However, it is unclear what types of cells secrete it and the action mechanism of SPC. To clarify them, the mechanisms surrounding the plasma membrane are important. Therefore, we investigated the action mechanisms of SPC around the cell membrane using fisetin, which prevents abnormal vascular contraction [14]. First, we investigated whether fisetin is directly bound to SPCs or functionally acted upon HCASMCs. When the action point of fisetin is SPC, the action of SPC should be inhibited and SPC-induced contraction should not occur. However, a premix of fisetin and SPC also resulted in SPC-induced contractions, similar to stimulations using SPC alone. Thus, we speculated that fisetin prevents SPC-induced contractions by interacting with HCASMCs directly and not by inhibiting SPC. This concurs with a previous report that fisetin and other flavonoids interact with membrane components [23]. These results showed that fisetin acted on HCASMCs directly and inhibited the function of SPC indirectly considered.

Next, we examined the involvement of microdomains such as lipid rafts and caveolae in SPC-induced contractions of HCASMCs as key parts of the plasma membrane and whether fisetin and SPC possibly act on the microdomains to regulate SPC-induced contraction. The results subsequently indicated that Flot1, a marker protein for lipid rafts, was more abundant in the microdomains of HCASMCs than Cav1, a marker protein for caveolae. Previous reports have suggested that caveolae are a lipid raft subtype [24,25]; therefore, the microdomains of HCASMCs were predominantly composed of lipid rafts. Moreover, to clarify the involvement of these microdomains, it was necessary to use microdomain-disrupted cells, in which cholesterol has been removed from the plasma membranes using MβCD. Consequently, both MβCD-treated and untreated cells exhibited SPC-induced contraction. Therefore, we concluded that microdomains are not related to the SPC-induced contraction of HCASMCs. Previous studies have also reported that SPC-induced contraction did not occur when cholesterol was removed from vascular tissue using MβCD [10,11]. One possible reason for this difference could have been that in vascular tissue, MβCD acts on the adjacent vascular endothelial cell layer rather than the vascular smooth muscle layer.

We then evaluated the localization of SPC to determine the initial reaction field of the plasma membrane. Since SPC has no fluorescence, we used commercially available NBD-SPC, which causes contractions similar to those caused by SPC. It has been reported that the hydrophilic group of NBD-SPC may be degraded by neutral sphingomyelinase in the plasma membrane to NBD-ceramide, which does not induce SPC-induced contraction [26], but there are no reports on the individual degradation of NBD or SPC. Notably, NBD-SPC was incorporated into HCASMCs regardless of fisetin treatment, and these results were also validated by NBD-SPC-positive cell rates using flow cytometry. Interestingly, as microdomain-disrupted cells were also positive for NBD-SPC, it is plausible that SPC did not interact with cholesterol. Nonetheless, further investigations are needed to verify these observations. Moreover, we speculated that fisetin does not prevent the incorporation of SPCs into HCASMCs, but functionally inhibits the action of SPCs incorporated into cells.

The SPC-stimulated increase of Flot1 in microdomains has suggested the new possibility of endocytosis being involved. Previous studies have reported that Flot1 activation induced exogenous endocytoses and that endocytosis may be either clathrin-dependent or microdomain/lipid raft/caveolae-dependent (Clathrin-independent); however, Flot1 is defined as a clathrin-independent endocytic pathway in mammalian cells [27,28,29,30]. Interestingly, the occurrence of microdomain-dependent endocytosis depends on Flot1 expression and is independent of Cav1 [30]. However, there have been no reports of endocytosis after SPC stimulation, and we examined the localization of SPCs. NBD-SPCs presented around the plasma membrane were incorporated into HCASMCs along with endosomes and subsequently remained intracellular, which was observed regardless of fisetin treatment. A previous study has reported that endocytosis of Flot1 is regulated by Fyn tyrosine kinase [31], which is activated in HCASMCs during SPC-induced contractions [9,32]. In addition, it was found that sphingoglycolipids underwent degradation in the plasma membrane; thus, we can assume this to be true for SPC as well. Therefore, SPC is very likely metabolized by Flot1-mediated endocytosis. Interestingly, microdomain-dependent endocytosis mechanisms also induced exocytosis to protect the plasma membrane from damage [33,34]. Endocytosis is a process involving the intake of extracellular molecules, whereas exocytosis involves extracellular secretion. Furthermore, this mechanism induces transcytosis in lung endothelial cells, an intracellular transport mechanism [35]. Thus, microdomain involvement has been implicated in endocytosis, exocytosis, and transcytosis. We further investigated the metabolism of SPC by assessing its role in transcytosis, i.e., transcytosis in the exocytosis of endocytic vesicles in cells. There are two types of SPCs attached to the membrane surface of HCASMCs, those that are incorporated into the cell and those that diverged from the cell surface, which generally occurs within 10 min after SPC stimulation. This is consistent with the fact that SPC-induced contraction is a progressive disease that takes only a few minutes [36]. It is noteworthy that SPCs remained in the cytoplasm during the entirety of our experiment, and no extracellular secretion of SPCs occurred, yet other endosomal vesicles, not containing SPCs, were released from HCASMCs. Indeed, we found that exosomes were produced by SPC-stimulated abnormally contracting cells, regardless of fisetin treatment. Since exosomes were also produced by non-contracting cells, their production was not involved in the preventive mechanism of fisetin. Exosomes are constantly being produced as part of the natural processes of cells [37], so they accumulate and increase in a time-dependent manner. In addition, exosomes play a role in intercellular communication [38]. However, the detailed relationship between exosomes and SPC-induced contraction has not been clarified, so further investigation is required to uncover this relationship.

## 5. Conclusions

In the present study, we found that fisetin prevents SPC-induced contractions by acting directly on HCASMCs without binding to SPC. Additionally, we established that cellular uptake of SPCs occurs via endocytosis, and SPCs are intracellularly metabolized. Endocytosis is triggered by microdomains, independent of the occurrence of SPC-induced contractions and the preventive mechanism of fisetin. Furthermore, although SPC-induced contracting cells produced exosomes, they did not leak SPCs. Thus, although HCASMCs take up SPCs via endocytosis and perform exocytosis, they do not expel SPC. Importantly, our finding that fisetin acts directly on HCASMCs is crucial to elucidate the preventive mechanism of fisetin in the future.

## Data Availability

The data presented in this study are available in the article or supplementary material.

## Supplementary Materials

The following supporting information can be downloaded at: https://www.mdpi.com/article/10.3390/cells12020265/s1, Figure S1: HCASMCs were not toxic by fisetin of less than 10 μM. Figure S2: Model diagram of SPR analysis. Binding quantity varies according to affinity. Figure S3: Fisetin prevents abnormal contraction of HCASMCs. Non-fisetin-treated cells are indicated by “SPC”. Arrows indicate contracted cells. Figure S4: Quantification of fisetin in cells or supernatant of fisetin-treated cells. Figure S5: Fluorescence image after induction of abnormal contraction by NBD-SPC.

## Abbreviations

Cav1: caveolin-1; Flot1: flotillin-1; HCASMCs: human coronary artery smooth muscle cells; MβCD: methyl-β-cyclodextrin; NBD: nitrobenzoxadiazole; PBS: phosphate-buffered saline; SPC: sphingosylphosphorylcholine; SPR: surface plasmon resonance.
