# Supplementary figures and images for: Sphingosylphosphorylcholine (SPC), a Causative Factor of SPC-Induced Vascular Smooth Muscle Cells Contraction, Is Taken Up via Endocytosis
Source: Cells. 2023 Jan 9;12(2):265. doi: 10.3390/cells12020265 (PMC9857160; doi:10.3390/cells12020265)

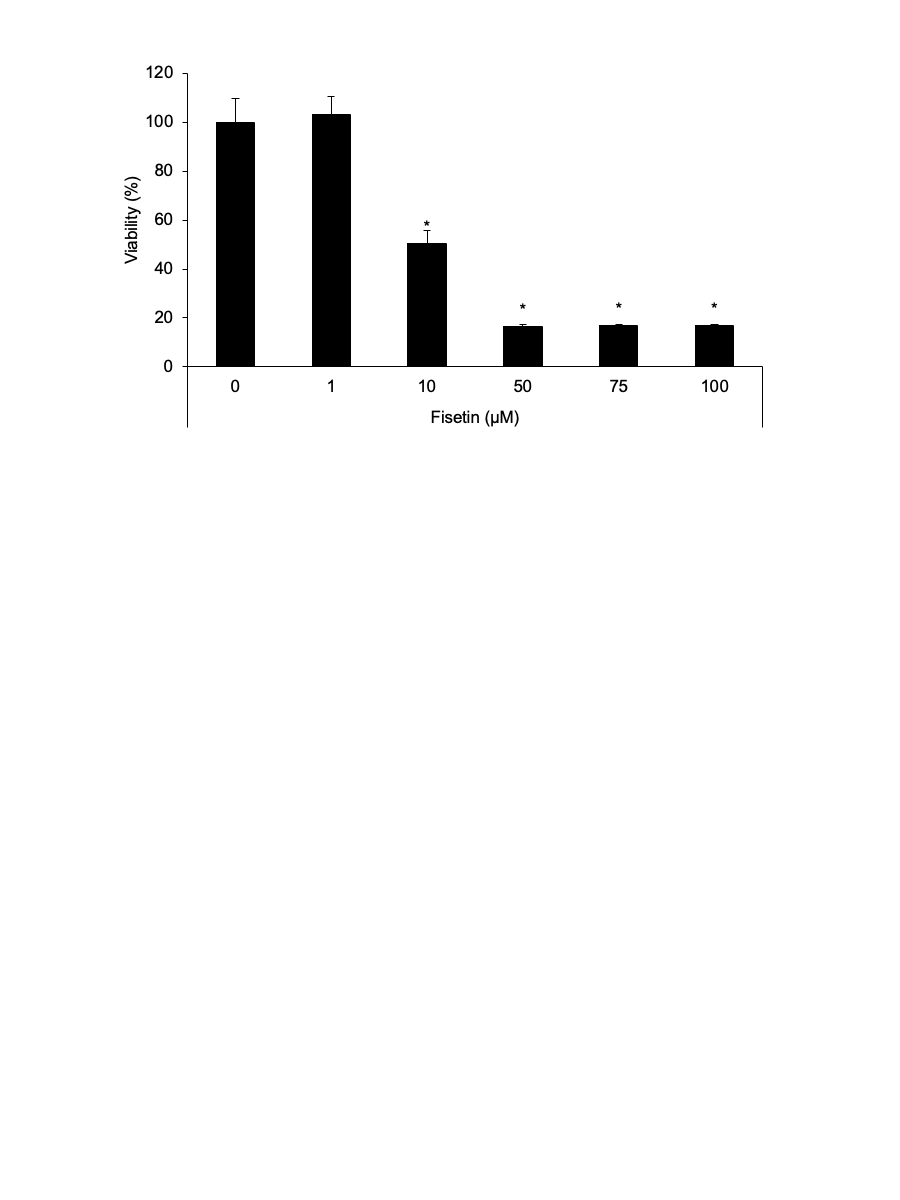

Supplement: Supplementary file 1 [file cells-12-00265-s001.zip › Supplement 1.tiff]

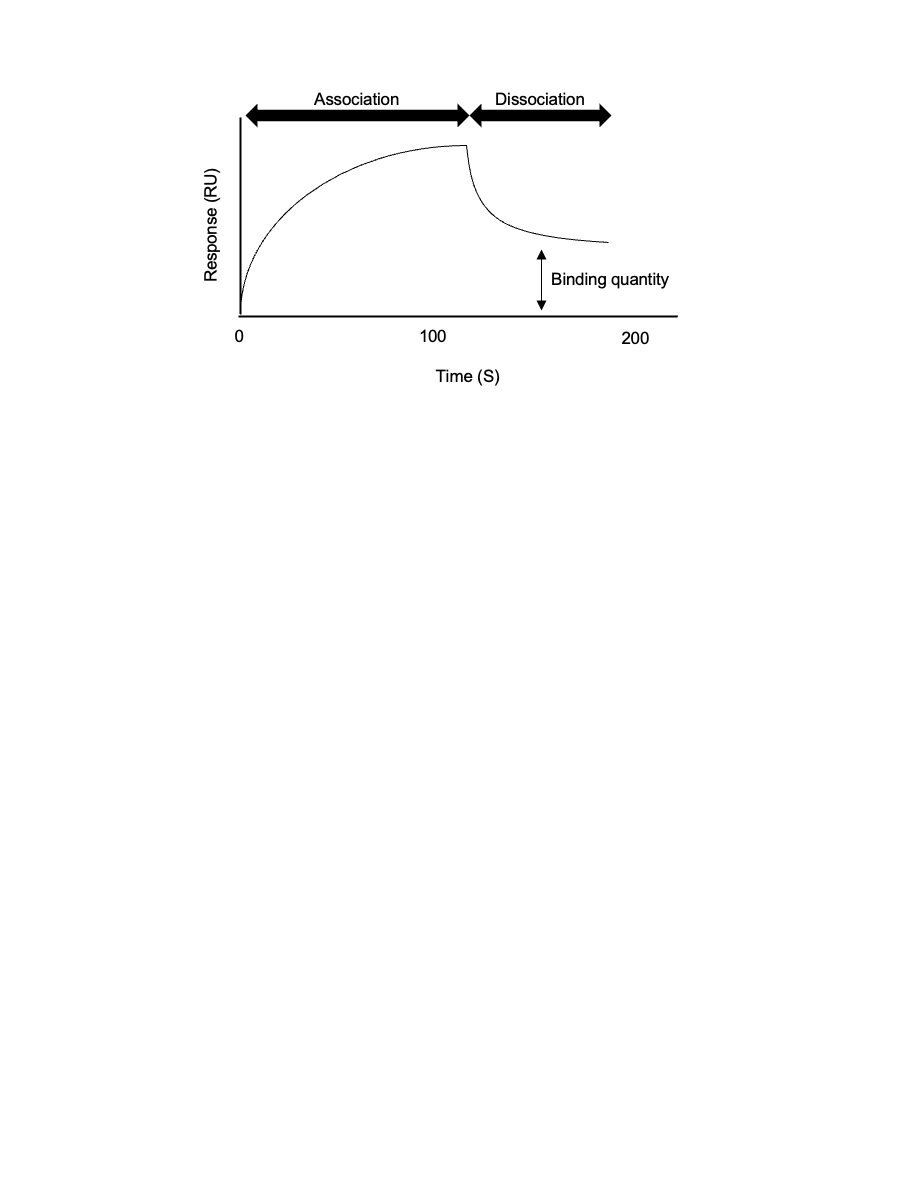

Supplement: Supplementary file 1 [file cells-12-00265-s001.zip › Supplement 2.tiff]

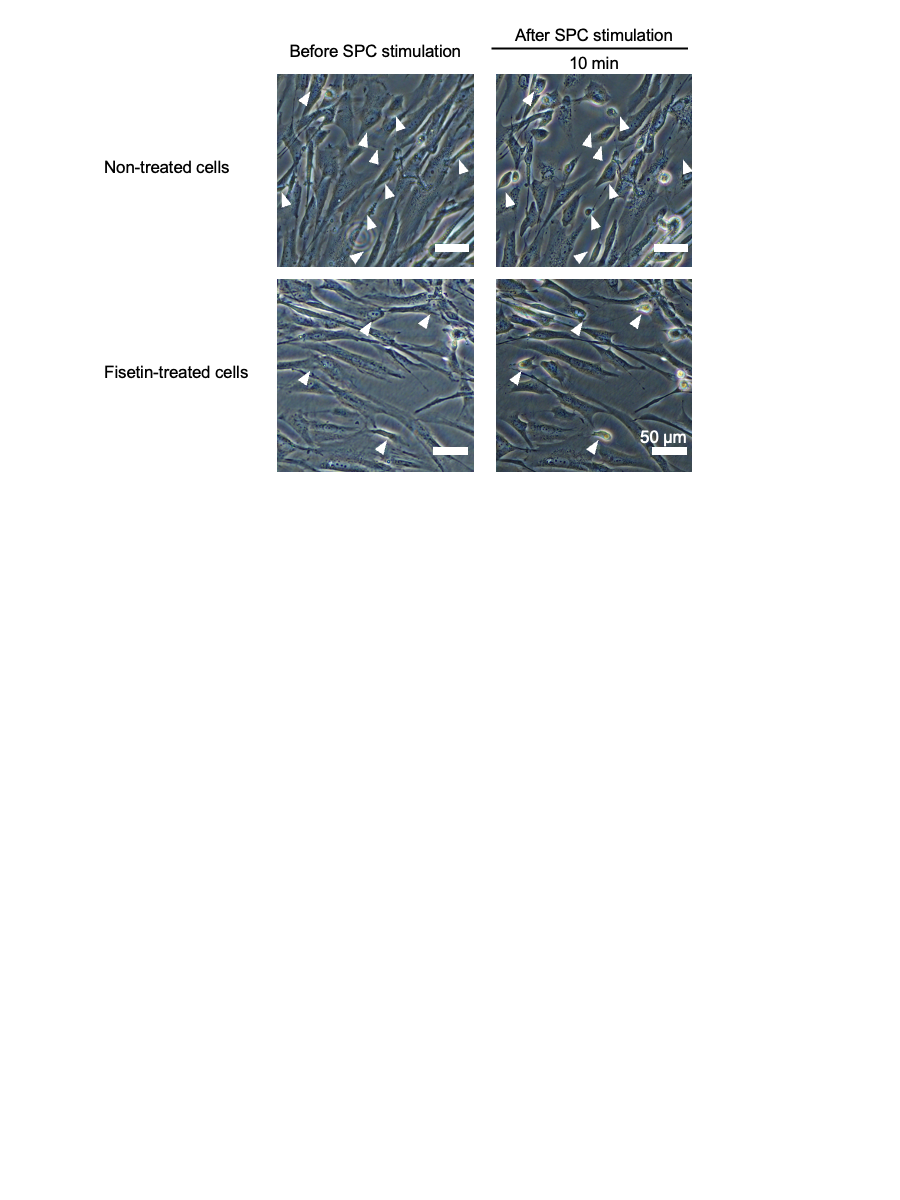

Supplement: Supplementary file 1 [file cells-12-00265-s001.zip › Supplement 3.tiff]

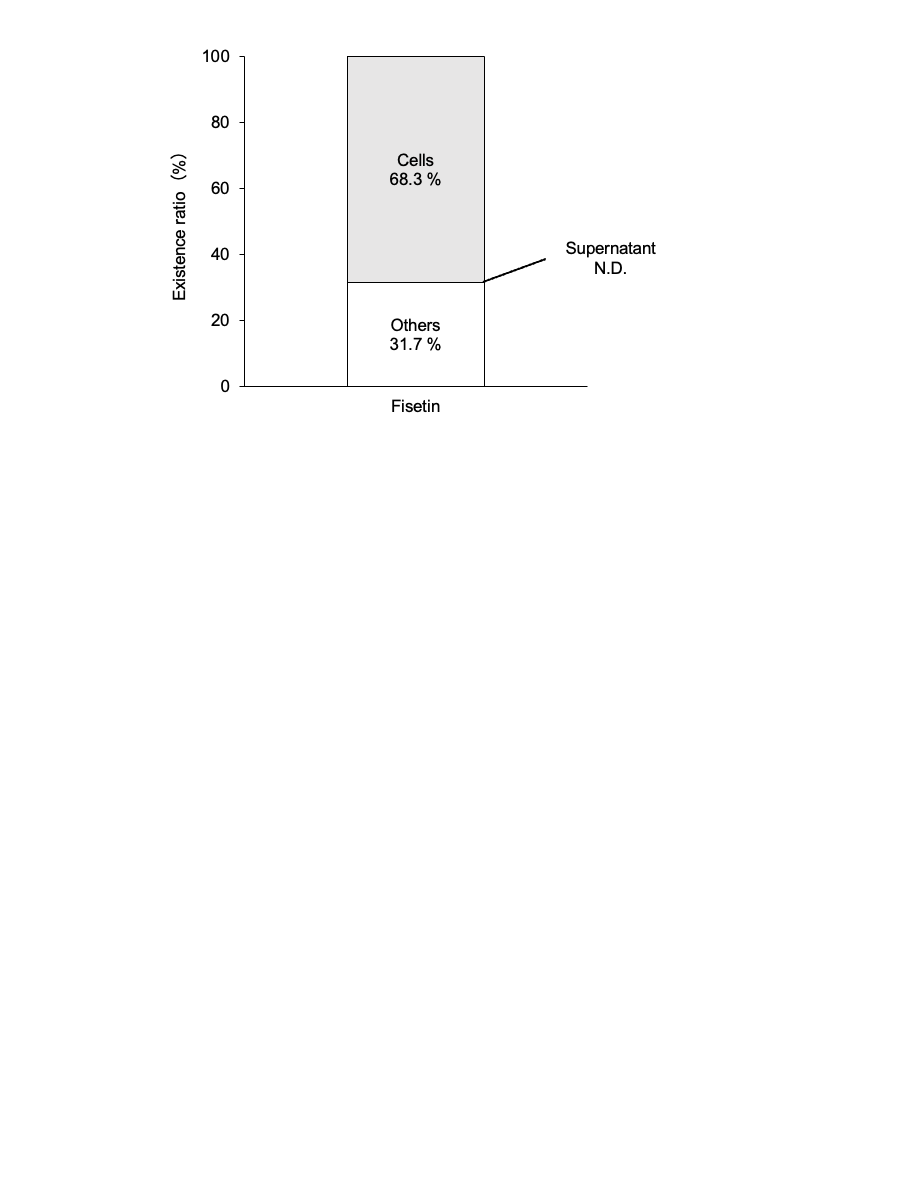

Supplement: Supplementary file 1 [file cells-12-00265-s001.zip › Supplement 4.tiff]

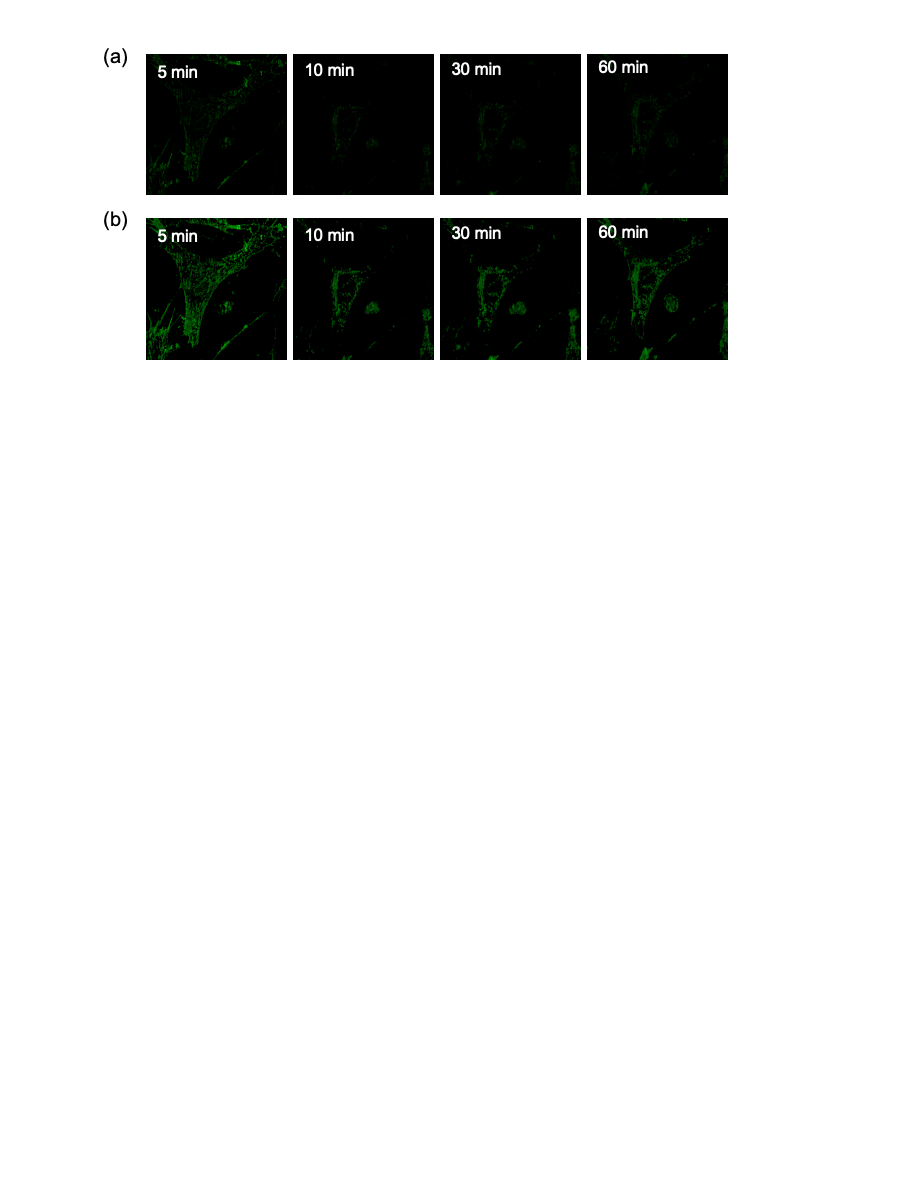

Supplement: Supplementary file 1 [file cells-12-00265-s001.zip › Supplement 5.tiff]
